# Adlay Seed (*Coix lacryma-jobi* L. var. *Ma-yuen* Stapf.) Ethanolic Extract Fractions and Subfractions Induce Cell Cycle Arrest and Apoptosis in Human Breast and Cervical Cancer Cell Lines

**DOI:** 10.3390/molecules27133984

**Published:** 2022-06-21

**Authors:** Yi-Fen Chiang, Cheng-Pei Chung, Jing-Hui Lin, Wenchang Chiang, Hsin-Yuan Chen, Mohamed Ali, Yin-Hwa Shih, Kai-Lee Wang, Tsui-Chin Huang, Hsin-Yi Chang, Li-Chun Lin, Tzong-Ming Shieh, Shih-Min Hsia

**Affiliations:** 1 School of Nutrition and Health Sciences, College of Nutrition, Taipei Medical University, Taipei 110301, Taiwan; yvonne840828@gmail.com (Y.-F.C.); hsin246@gmail.com (H.-Y.C.); lichun.lin@tmu.edu.tw (L.-C.L.); 2 Department of Nutrition and Health Sciences, Chang Gung University of Science and Technology, Taoyuan 33303, Taiwan; cpchung@mail.cgust.edu.tw; 3 Research Center for Food and Cosmetic Safety, Chang Gung University of Science and Technology, Taoyuan 33303, Taiwan; 4 Research Center for Chinese Herbal Medicine, College of Human Ecology, Chang Gung University of Science and Technology, Taoyuan 33303, Taiwan; 5 College of Bioresources and Agriculture, National Taiwan University, Taipei 10617, Taiwan; brightgirl3310@yahoo.com.tw (J.-H.L.); chiang@ntu.edu.tw (W.C.); 6 Clinical Pharmacy Department, Faculty of Pharmacy, Ain Shams University, Cairo 11566, Egypt; mohamed.aboouf@pharma.asu.edu.eg; 7 Department of Healthcare Administration, Asia University, Taichung 41354, Taiwan; evashih@asia.edu.tw; 8 Department of Nursing, Ching Kuo Institute of Management and Health, Keelung City 203301, Taiwan; kellywang111@gmail.com; 9 Graduate Institute of Cancer Biology and Drug Discovery, Taipei Medical University, Taipei 11031, Taiwan; tsuichin@tmu.edu.tw; 10 Graduate Institute of Medical Sciences, National Defense Medical Center, Taipei 11490, Taiwan; hsinyi.chang@mail.ndmctsgh.edu.tw; 11 School of Dentistry, College of Dentistry, China Medical University, Taichung 404333, Taiwan; 12 Nutrition Research Center, Taipei Medical University Hospital, Taipei 110301, Taiwan; 13 School of Food and Safety, Taipei Medical University, Taipei 11031, Taiwan; 14 Graduate Institute of Metabolism and Obesity Sciences, Taipei Medical University, Taipei 11031, Taiwan

**Keywords:** adlay seed, breast cancer, cervical cancer, caspase-3, apoptosis, cell cycle

## Abstract

The antitumor effects of *Coix lacryma-jobi* L. var. ma-yuen Stapf. (adlay seed) ethanolic extract have been increasingly shown. This study aimed to investigate the beneficial effects of both the fractions and subfractions of adlay seed ethanolic extract on the human breast (MCF-7) and cervical (HeLa) cancer cell lines, as well as exploring their possible mechanisms of action. The ethanolic extracts were obtained from different parts of adlay seed, including AHE (adlay hull extract), ATE (adlay testa extract), ABE (adlay bran extract) and PAE (polished adlay extract). The results of a 3-(4,5-dimethyl thiazol-2-yl)-2,5-diphenyl- tetrazolium bromide (MTT) assay showed that AHE-Ea and ATE-Ea showed significant growth inhibitory effects in a dose-dependent manner. The results also showed that the AHE-Ea-K, AHE-Ea-L, ATE-Ea-E and ATE-Ea-F subfractions inhibited cell proliferation, induced cell cycle arrest in the G0/G1 phase and decreased CDK4/Cyclin D1 protein expression. Finally, the extract activated caspase-3 activity and PARP protein expression, which induced MCF-7 and HeLa cell apoptosis. We then used liquid chromatography–mass spectrometry (LC/MS) to identify the potential active components., Quercetin showed an anticancer capacity. In conclusion, the AHE-Ea-K, AHE-Ea-L, ATE-Ea-E and ATE-Ea-F subfractions showed antitumor effects through the inhibition of MCF-7 and HeLa cell line viability, as well as inducing apoptosis and cell cycle arrest.

## 1. Introduction

Cancer is among the most debilitating diseases worldwide. In women, breast cancer (including metastasis) and gynecological malignancies are the leading causes of death. In 2018, more than two million cases of breast cancer were diagnosed and mortality was estimated to be around 600,000 deaths, while in cervical cancer, the number of new cases was 560,000 and there were 311,000 deaths worldwide [1]. Notably, breast cancer accounts for the highest incidence and mortality rates out of all of the different types of cancers in women. In female-related gynecological cancers, cervical cancer has the fourth highest incidence and mortality rates [2]. Cancer is globally believed to be a disease of abnormal cell differentiation [3]. Ideally, effective anticancer treatment would suppress cancer via the induction of cancer cell apoptosis, which in turn would preclude their hazardous effects on surrounding normal cells by creating inflammation microenvironments [4,5]. Caspase activation is one of the main hallmarks of apoptosis induction [6], especially caspase-3, which is the most important executor caspase. The characteristics of apoptosis, such as DNA fragmentation, nuclear condensation and the formation of cell bodies, are all dependent on the activation of caspase-3 [7]. 

Traditional Chinese medicine for cancer has been increasingly studied and has shown different anticancer mechanisms, including cell apoptosis induction and cell viability inhibition [8]. Coix is one of the major compounds that is currently being utilized in the research and development of health foods. It is an annual herbaceous crop from the Gramineae family and its full name is Coix lacryma-jobi L. var. ma-yuen Stapf. Adlay seeds are suitable for planting in Taiwan and the crops are grown in paddy fields. Coix has been among the first-class materials used as medicinal and dietary supplements since ancient times. Recent studies have also pointed out that Coix has antitumor [8], anti-allergic [9], hypolipidemic [10], hypoglycemic [11], anti-oxidant [12] and ovulation-inducing effects [13]. 

In this study, we explored the antitumor effects of adlay ethanolic extract, as well as its subfractions, on the most common female cancers, including breast and cervical cancers, through the measurement of cell proliferation, cell cycle and apoptosis.

## 2. Results

### 2.1. Effects of Different Parts of the Adlay Seed and Its Ethanolic Extract Fractions on Cancer Cell Viability 

**Figure 1:**
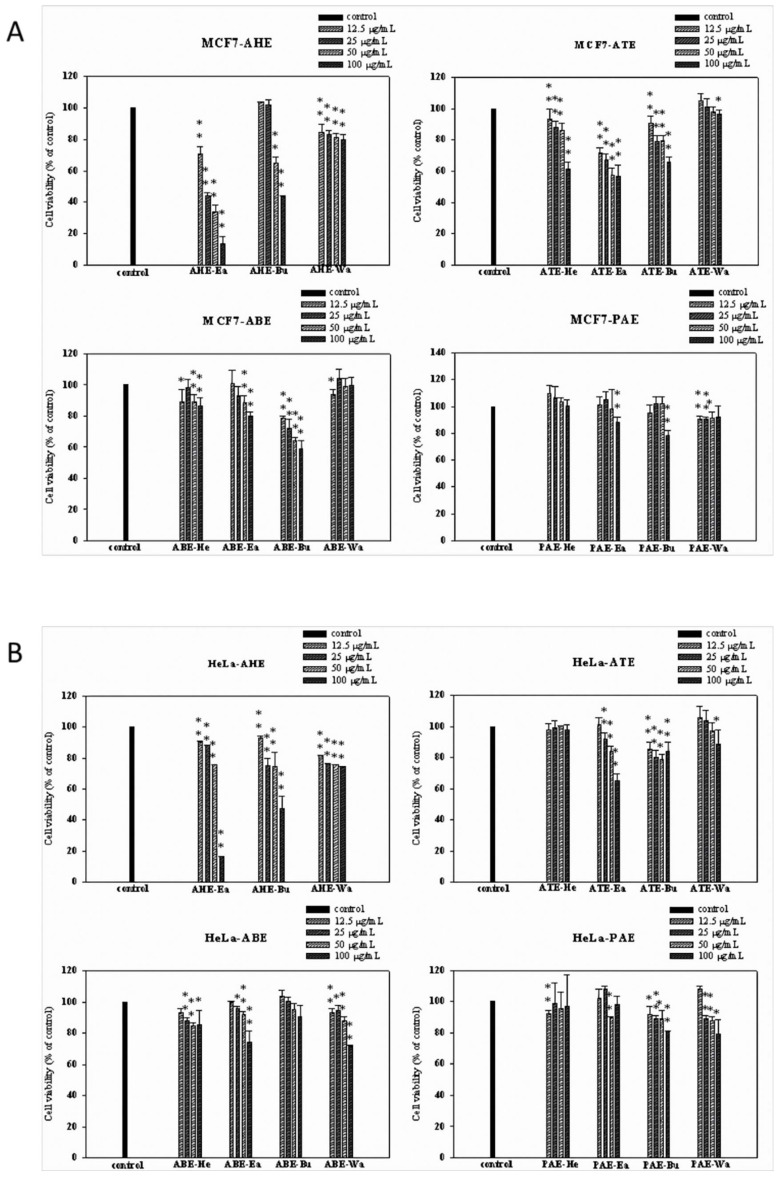
The antiproliferative effects of different fractions from various adlay seed ethanolic extracts on (**A**) MCF-7 and (**B**) HeLa cells. The MCF-7 and HeLa cells (2 × 10^3^ cells/well) were cultured with or without treatment (0, 12.5, 25, 50 and 100 μg/mL) for 72 h and cell viability was measured using an MTT assay. The results were expressed as a percentage of living cells cultured in the presence of treatment compared to the untreated control. Each bar represents the mean ± SD (*n* = 3). The statistical significance was measured using Student’s t-tests: * *p* < 0.05 compared to the control group; ** *p* < 0.01 compared to the control group (con, control; He, hexane; Bu, butanol; Wa, water).

Different dosages of adlay seeds and extracts from four parts of the plant (adlay hull (AHE), testa (ATE), bran (ABE) and polished adlay (PAE)) were tested. Treatment with AHE-Ea, ATE-Ea, ATE-Bu and ABE-Bu that started from 12.5 μg/mL for 72 h significantly inhibited MCF-7 cell viability in a dose-dependent manner. Moreover, fractionation that was based on polarity resulted in the subfractions having different effects. All subfractions showed antiproliferative effects on MCF-7 cell except ATE-Wa, ABE-Wa, PAE-He, PAE-Ea and PAE-Bu. (Figure 1A). 

As for effects on HeLa cells, all fractions showed significant growth inhibitory effects at 100 μg/mL concentrations. Notably, the ATE-Ea, ABE-Ea and ABE-Wa subfractions showed promising antiproliferative effects in a dose-dependent manner, unlike ATE-He, ABE-Bu, PAE-He and PAE-Ea (Figure 1B). 

### 2.2. Effects of Different Subfractions of Adlay Hull and Testa Extracts on Cancer Cell Viability

**Figure 2:**
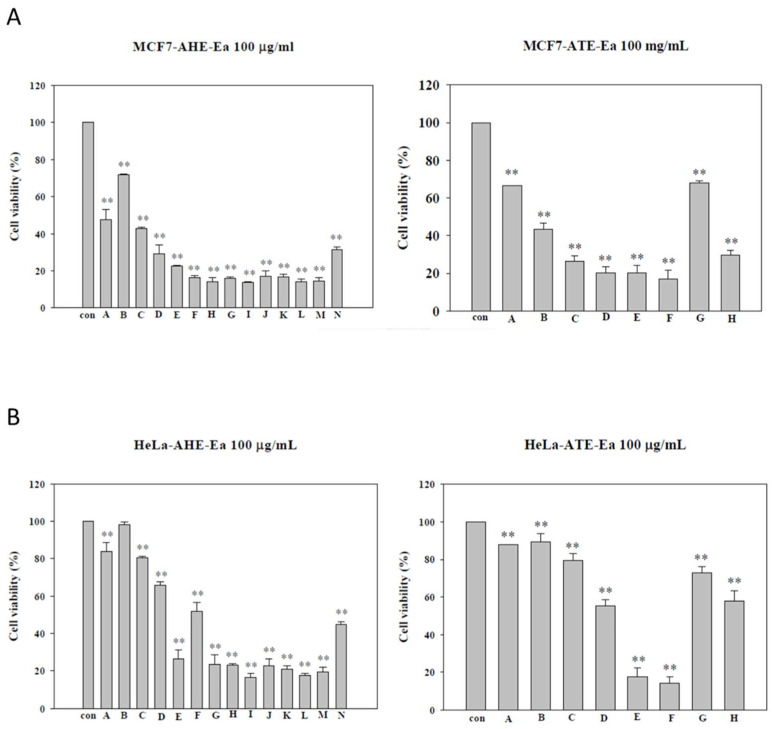
The effects of the AHE-Ea and ATE-Ea subfractions on the cell viability of (**A**) MCF-7 and (**B**) HeLa cells. The MCF-7 and HeLa cells (2 × 10^3^ cells /well) were cultured with or without treatment (100 μg/mL) for 72 h and cell proliferation was measured using an MTT assay. The results were expressed as a percentage of living cells cultured in the presence of treatment compared to the untreated control. Each bar represents the mean ± SD (*n* = 3). The statistical significance was measured using Student’s t-tests: * *p* < 0.05 compared to the control group; ** *p* < 0.01 compared to the control group.

Since AHE and ATE showed the most antiproliferative effects on cancer cell lines, we further explored the effects of their subfractions. The AHE and its fourteen subfractions were obtained according to the similarity of each collection by thin layer chromatography (TLC). In MCF-7 cells (Figure 2A), the AHE-Ea-(A–N) subfractions significantly decreased MCF-7 cell viability with IC50 ranging from 13.7 to 146.3 μg/mL (Table 1). In HeLa cells (Figure 2B), aside from the AHE-Ea-(A–C) subfractions, all of the subfractions had significant growth reduction effects. As for the ATE and its eight subfractions, they significantly decreased MCF-7 cell viability with IC50 ranging from 13.7 to 146.3 μg/mL (Table 2). Similar effects were demonstrated in HeLa cells with IC50 ranging from 13.7 to 146.3 μg/mL (Table 2) and the subfraction ATE-Ea-F showing the best growth inhibition effects (Figure 2B).

### 2.3. Effects of AHE-Ea-K, AHE-Ea-L, ATE-Ea-E and ATE-Ea-F Subfractions on Cancer Cell Cycle Distribution 

Following the MTT results, the AHE-Ea-K, AHE-Ea-L, ATE-Ea-E and ATE-Ea-F subfractions showed the best growth inhibitory effects on cancer cell lines. Therefore, we further explored their effects on cancer cell cycle distribution using flow cytometry in both MCF-7 and HeLa cells. Different concentrations of AHE-Ea-K and AHE-Ea-L (0, 25, 50 and 75 μg/mL) were used for 48 h. The treatments resulted in the significant cell cycle arrest of MCF-7 cells in the Go/G1 phase (Table 3) and HeLa cells (Table 3) in the S phase. The ATE-Ea-E and ATE-Ea-F treatments of 0, 25, 50 and 75 μg/mL for 48 h caused the significant cell cycle arrest of MCF-7 cells and HeLa cells in the G0/G1 phase (Table 3). 

### 2.4. Effects of AHE-Ea-K, AHE-Ea-K L, ATE-Ea-E and ATE-Ea-F on Caspase-3 Activity

**Figure 3:**
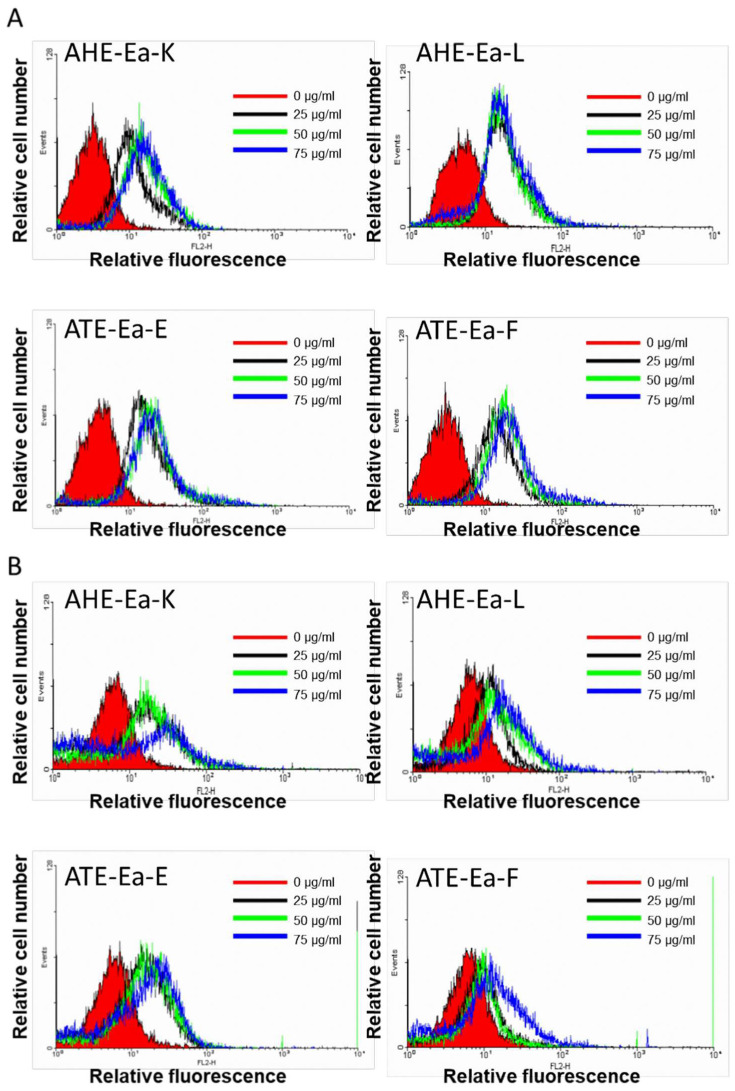
The effects of adlay extract subfractions on the activity of caspase-3 in (**A**) MCF-7 or (**B**) HeLa cells. The MCF-7 and HeLa cells were cultured in MEM and DMEM media containing 0, 25, 50 and 75 μg/mL of AHE-Ea-K, AHE-Ea-L, ATE-Ea-E or ATE-Ea-F for 48 h. Then, the cells were stained using a caspase-3 kit and analyzed by flow cytometry. Each data point represents as the mean ± SD (*n* = 3).

Since activation and cleavage of caspase-3 is considered to be the key modulator in cell apoptosis [6], we used flow cytometry to explore the changes in caspase-3 activity in cancer cell lines following treatment with 75 or 100 μg/mL of AHE-Ea-K, AHE-Ea-K L, ATE-Ea-E or ATE-Ea-F for 48 h. The treatments significantly increased the caspase-3 activity, as shown by the strengthening of the relative fluorescence signal (Figure 3), as well as quantitation (Table 4). 

### 2.5. Anticancer Properties of Adlay Extract and its Active Components

**Figure 4:**
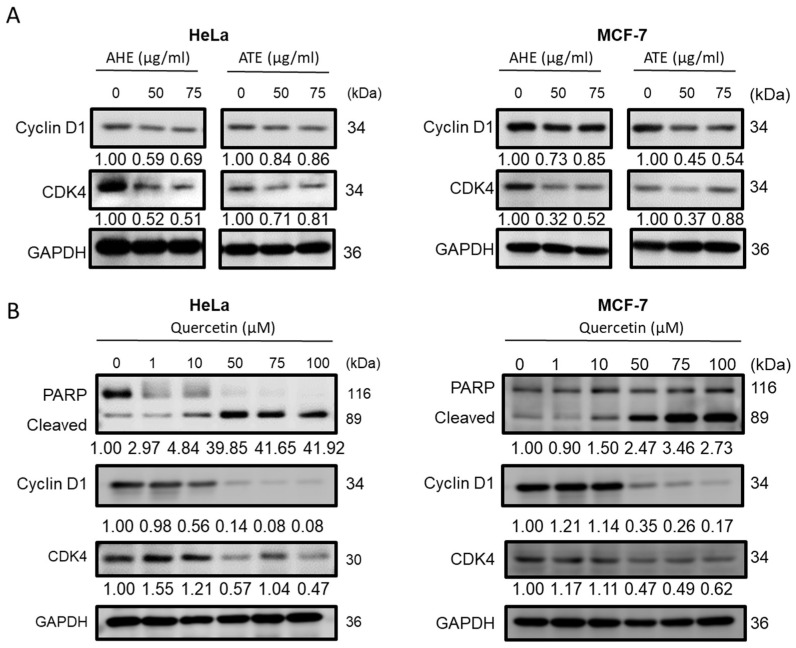
The effects of adlay extracts or the active component quercetin on cell cycle arrest and apoptosis-related protein expression in HeLa and MCF-7 cells. The HeLa or MCF-7 cells were cultured in DMEM and MEM media containing (**A**) AHE or ATE or (**B**) quercetin for 48 h. Then, a western blot was used for the protein expression analysis.

To explore the anticancer effects of AHE and ATE on Hela and MCF-7 cells, the cells were treated with 50 or 75 μg/mL of ATE or AHE for 48 h. The ATE and AHE treatment could successfully suspend the cyclin D1 and CDK4 protein expression (Figure 4A). To explore the potential components in the adlay extracts, the results of the LC-MS analysis of the combinations of active subfractions from AHE-Ea and ATE-Ea are shown in the Supplementary Material, Figure S1. According to their inhibitory activities on HeLa and MCF-7 (Table 1 and Table 2), AHE-EA-E–L, ATE-EA-E and ATE-Ea-F were combined to be regarded as active subfractions. Among the compounds that were isolated from the Coix seeds, naringenin, quercetin and vanillin were identified in the sum intensity of the total ion chromatogram (TIC) of the AHE-Ea active combined subfraction. In comparison to AHE-Ea, ATE-Ea-E and ATE-Ea-F contained more phenolic compounds, including quercetin, luteolin, apigenin, homoeriodictyol, eriodictyol, naringenin, isoliquiritigenin, chrysoeriol and vanillic acid. Based on the LC-MS analyses, quercetin and naringenin both existed in the active combined subfractions from AHE and ATE, which means that these two compounds could play important roles in anticancer properties. As shown in Figure 4B, quercetin increased the PARP and decreased CDK4 and Cyclin D1 protein expression, which led the apoptosis progression and caused the cell cycle arrest in the G0/G1 phase when treated for 48 h. Otherwise, naringenin did not show any significant activity under the same dosages as quercetin (data not shown).

## 3. Discussion

Coix seeds can be divided into four parts from the outside to the inside, namely the Coix husk, Coix seed coat, Coix bran and refined white Coix seed. These four parts can be extracted using ethanol to obtain Coix husk ethanol extract (AHE), Coix seed coat ethanol extract (ATE), Coix bran ethanol extract (ABE) and polished Coix seed ethanol extract (PAE). These four ethanol extracts are then divided into the n-hexane layer (He), ethyl acetate layer (Ea), n-butanol layer (Bu) and water layer (Wa) using solvents of different polarities [12]. Following the use of the MTT analysis method to explore the antiproliferative effects of these test samples at different concentrations (0, 12.5, 25, 50 and 100 μg/mL) on breast cancer cells (MCF-7) and cervical cancer cells (HeLa) for 72 h, the results revealed that the most effective subfractions were those extracted from AHE and ATE. In a previous study, the treatment of blood cancer cells HL-60 and U937 with a methanol extract of Coix shell (AHM) for 72 h significantly inhibited cell growth with IC50 values of 70 and 61 μg/mL, respectively. Additionally, adlay seed extracts did not show any significant cytotoxicity on the normal human lung fibroblast MRC-5 cell line, which means that the test sample had no cytotoxicity [14]. In a tobacco-specific carcinogen 4-(methylnitrosamino)-1-(3-pyridyl)-1-butanone (NNK)-induced lung tumorigenesis animal model, feed with 30% adlay seed could reduce the number of lung tumors [15], indicating the anticancer ability of adlay seeds on various cancers. 

Using a rapid proliferation cell model, we previously showed that the ethyl acetate fraction of adlay hull (AHE-Ea) could inhibit the growth of leiomyoma [16]. Notably, different layers had different effects. The hexane layer of the adlay testa ethanolic extract (ATE-Hex) could enhance the chemo-sensitivity and inhibit the growth of human uterine sarcoma cancer cells via apoptosis induction [17]. The AHE-Ea and ATE-Ea extracts had stronger growth inhibitory effects on various cancer cell lines. The two differentiating layers were chromatographed using open silica gel-filled columns and the AHE-Ea was divided into the A–N layers and ATE-Ea was divided into the A–H layers. An MTT assay was further used to investigate their cell growth inhibition abilities. An ethyl acetate extract of the traditional Chinese medicine Patrinia scabiosaefolia on the proliferation of MCF-7 cells showed great growth inhibitory effects with an IC50 value of 112.3 μg/mL [18]. Moreover, a previous study demonstrated that MCF-7 cells that were treated with the ethyl acetate extract of shiitake mushrooms at a concentration of 50 μg/mL had a 50% increase in apoptosis [19]. 

Identifying the potential active components in adlay extracts may need further study. However, several studies have indicated that the components in AHM that inhibit the proliferation of blood cancer cells or have cytotoxicity effects may be syringaldehyde, syringaresinol, tricin, ω-hydroxypropioguaiacone and naringenin [14]. Jeong et al. [19] also pointed out that syringaresinol and tricin can inhibit the growth of MCF-7 cells, as well as naringenin, with an IC50 value of 287 nM [20]. Further study is needed to explore whether these three pure substances are responsible for the growth inhibitory effects of AHE-Ea on MCF-7 cell lines. In addition, Guo et al. [14] and Xie et al. [21] showed that there are 17 kinds of antitumor ingredients in Coix, including three kinds of phenolic aldehydes (vanillin, etc.), p-hydroxybenzaldehyde, syringaldehyde, one phenolic ketone (ω-hydroxypropioguaiacone), four phenolic acids (trans-p-coumaric acid, syringic acid, ferulic acid and vanillic acid), two flavonoids (naringenin and tricin), three kinds of fatty acids (oleic acid, linoleic acid and linolenic acid), β-sitosterol, trypsin inhibitor, α-monolinolein and coixenolide. In the present study, several other flavonoids in AHE and ATE were further identified by LC-MS (Supplementary Material, Figure S1), such as quercetin, luteolin, apigenin, homoeriodictyol, eriodictyol, naringenin, isoliquiritigenin and chrysoeriol. Our results revealed that adlay hull and testa are rich in flavonoids.

Following our previous study using HPLC and GC/FID, ATE-EA was found to contain phenolic compounds, including protocatechuic acid, p-hydroxybenzoic acid, chlorogenic acid, vanillic acid, p-hydroxybenzaldehyde, syringic acid, vanillin, syringaldehyde, caffeic acid, p-coumaric acid and ferulic acid. Therefore, ATE-EA showed promising antitumor effects on endometrial cancer cells [8]. The effects of AHE-Ea-K, AHE-Ea-L, ATE-Ea-E and ATE-Ea-F on MCF-7 cells could be related to the presence of active flavonoids in the effective subfraction layer, such as naringenin. This substance can compete with estradiol (17β-estradiol) for estrogen receptor β and thus, exerts its anti-estradiol effects [22]. Since MCF-7 is a breast cancer cell line that expresses the estrogen receptor β, this could explain the mechanisms of the antitumor effects of these subfractions on MCF-7 cells. In the present study, quercetin was proven to induce cell apoptosis and cell cycle arrest (Figure 4) and be one of the important anticancer compounds in AHE and ATE.

Anticancer effects not only cause the induction of cell cycle arrest but also apoptosis activation [23]. Our results showed that adlay ethanolic extract subfractions significantly induced cell cycle arrest, as well as the induction of caspase 3 activation-mediated apoptosis.

## 4. Materials and Methods

### 4.1. Adlay Extract Preparation 

**Figure 5:**
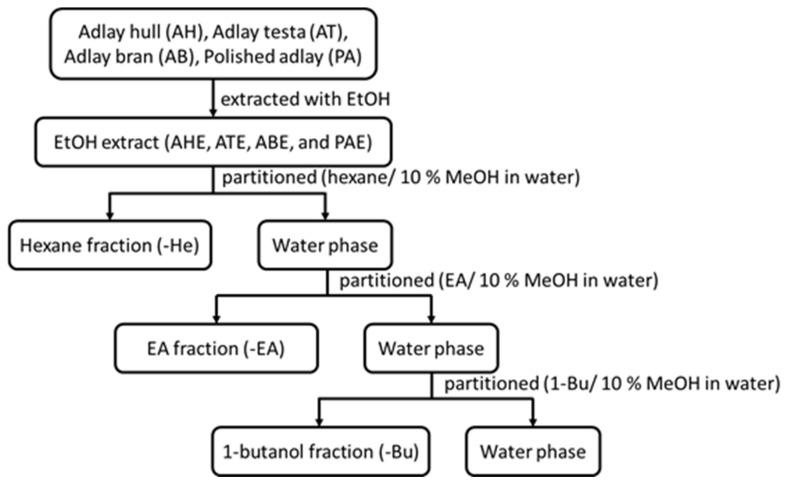
The solvent partitions of the ethanolic extracts from different parts of the adlay seeds.

The Coix seeds were processed by a dehulling machine to obtain the outer shell (35.8% of the weight of the Coix seeds) and then the skin and Coix seeds were air-sieved to obtain the testa (6.8% of the weight of the Coix seeds). The Coix seeds were further divided into Coix bran (4.3% of the weight of the Coix seeds) and refined white Coix seed (53.1% of the weight of the Coix seeds) [8]. Dehulled adlay, consisting of bran and endosperm, is regarded as a healthy cereal material, while the adlay hull and testa are treated as waste. The extracts were first extracted using 90% ethanol and then they were dried, resuspended in 10% methanol (aq.) and partitioned with 1:1 volume n-hexane until they were colorless. Then, they were dried under a vacuum to obtain the n-hexane fraction (He). The aqueous layer was further partitioned using ethyl acetate (EA) and n-butanol (Bu) to obtain the next two fractions (Figure 5) [8].

The EA fractions of the different parts of the Coix seeds (bran and testa) were eluted using silica gel open column chromatography (OCC) with gradually increasing proportions of He or EA, from 0% to 100%, and were then eluted using methanol. The eluents were expanded with thin-layer chromatography (TLC). Subfractions with similar chemical compositions from the TLC were combined to obtain AHE-EA-A–N and ATE-EA-A–H.

After the fractions were air-dried, DMSO was used as a solvent to make the 100 mg/mL of stock solution for further experiments.

### 4.2. Cell Line and Culture Conditions

HeLa (human cervix adenocarcinoma cell line) and MCF-7 (human breast adenocarcinoma) cells were obtained from the Bioresource Collection and Research Center (BCRC, Hsinchu, Taiwan). The cells were maintained in 10% fetal bovine serum (FBS, Biological Inc., Kibbutz Beit-Haemek, Israel) and supplemented with 1% penicillin–streptomycin–amphotericin B solution in Dulbecco’s modified Eagle’s medium (DMEM, BRL Gibco Inc., Grand Island, NY, USA) or in minimum essential medium (MEM) in a humidified incubator (Asheville NC, North Carolina, USA (37 °C; 5% CO_2_)).

### 4.3. MTT Assay

The cells were planted in 96-well plates (2 × 10^3^ cells/well) and treated with different concentrations (12.5, 25, 50, 100, 200 μg/mL) of the adlay seed extracts. The cell viability was analyzed using an MTT assay (3-(4,5-dimethyl-2-thiazolyl)-2,5-diphenyl-2H-tetrazolium bromide; Abcam, Cambridge, MA, USA). At the end of the treatment, a serum-free medium with 1 mg/mL of MTT was substituted by the conditional medium and incubated for an additional 4 h. Subsequently, the media were removed. The crystal formazan was dissolved by in 100 μL/well of dimethyl sulfoxide (DMSO; ECHO Chemical Co. Ltd., Taipei, Taiwan). The optical density was measured using a VERSA Max microplate reader (Molecular Devices, San Jose, CA, USA) at 570 nm and 630 nm as the reference wavelength.

### 4.4. Cell Cycle Distribution 

To analyze the cell cycle distribution, the cells (3 × 10^5^) were planted into 6-well plates. After attaching overnight, the cells were treated with the AHE-Ea-K, AHE-Ea-L, ATE-Ea-E and ATE-Ea-F extracts in a culture medium containing 10% FBS for 48 h. At the end of the incubation, the cells were detached using trypsin. The cells were fixed in 70% alcohol at −20 °C and stained with a propidium iodide (PI) solution (2 mg of DNAse-free RNAse A and 0.4 mL of 500 μg/mL PI added to 10 mL of 0.1% Triton X-100 in PBS; Sigma-Aldrich, St. Louis, MO, USA) at room temperature for 30 min. Then, using a BD FACSCanto flow cytometer (BD Biosciences, San Jose, CA, USA), a minimum of 10,000 cells per sample was collected and further analyzed using CellQuest software (BD Biosciences). 

### 4.5. Caspase-3 Activity Analysis 

The cells were planted in 6-well plates (3 × 10^5^ cell/well) and treated for 48 h. The cells were then trypsinized to detach the cell and then, we added the caspase-3-specific fluorescence reagent PhiPhiLuxTM–G2D2 (Millipore, Billerica, MA, USA) for 1 h at 37 °C, following the manufacturer’s instructions. We used flow cytometry to capture the fluorescence signals. The signals were quantified and compared to the untreated control and unstained groups. 

### 4.6. Protein Extraction and Western Blot Analysis

The cells were lysed in an RIPA lysis buffer containing protease and phosphatase inhibitors (Roche, Mannheim, Baden-Württemberg, Germany). The protein was quantitated using a bicinchoninic acid (BCA) assay and sodium dodecyl sulfate polyacrylamide gel electrophoresis (SDS-PAGE) with 95 voltages and a 2 h transfer to a polyvinylidene fluoride (PVDF) membrane. We used a 5% bovine serum albumin (BSA) solution to block for 1 h. Subsequently, the membranes were incubated with the primary antibodies CDK4 (1:1000; Abcam, Cambridge, UK), cyclin D1 (1:1000; Cell Signaling, Boston, MA, USA), PARP (1:1000; Cell Signaling) and GAPDH (1: 10,000; Proteintech, Rosemont, IL, USA) at 4 °C overnight and then with a horseradish peroxidase (HRP)-conjugated secondary antibody (1: 10,000) for 1 to 2 h. The signals were captured by an eBlot Touch Imager (eBlot Photoelectric Technology, Shanghai, China). The band densities were determined using the Image J software program, version 1.52 (NIH, Bethesda, MD, USA). The expression levels of these target proteins were analyzed in three individual experiments. 

### 4.7. Statistical Analysis

All data are presented as the mean ± SD and the MTT results were analyzed using Student’s t-tests. The statistical significance was presented by *p*-values of less than 0.05 and *p*-values of less than 0.001. The flow cytometry results were analyzed using one-way analysis of variance and Duncan’s new multiple range tests to examine the differences between the treatments using the statistical package for social science (SPSS) software. 

## 5. Conclusions

We utilized an in vitro cell model to investigate the antitumor effects of ethanolic extracts from the differentiating layers and subfractions of the different parts of Coix seeds on breast and cervical cancer cell lines. AHE-Ea and ATE-Ea showed promising results. Moreover, among the subfraction layers, the medium- and high-polarity subfraction layers could effectively inhibit cancer cell growth through cell cycle arrest, activate caspase-3 protein and eventually induce cell apoptosis. Active subfractions were prepared and the compounds that were isolated from the AHE and ATE were analyzed by LC-MS. Among these identified compounds, quercetin showed significant anticancer activity and could be one of the important anticancer components in AHE and ATE.

## Figures and Tables

**Table 1:** The IC50 of the AHE-Ea subfractions in MCF-7 and HeLa cells.

AHE-Ea	MCF-7 (μg/mL)	HeLa (μg/mL)
A	100.5	>200
B	146.3	>200
C	81.9	>200
D	43.8	129.2
E	34.8	38.7
F	31.4	108.3
G	14.2	49.63
H	14.2	47.7
I	14.4	34.7
J	32.9	38.2
K	13.7	32.4
L	13.7	38.4
M	21.2	98.8
N	60.9	1.8

The MCF-7 or HeLa cells (2 × 10^3^ cells /well) were cultured with different AHE-Ea subfractions (6.25, 12.5, 25, 50 and 100 μg/mL) for 72 h and the IC50 was calculated from the measurement of cell proliferation using an MTT assay.

**Table 2:** The IC50 of the ATE-Ea subfractions in MCF-7 and HeLa cells.

ATE-Ea	MCF-7 (μg/mL)	HeLa (μg/mL)
A	121.7	151.9
B	75.7	145.4
C	46.4	133.3
D	54.0	114.8
E	25.0	58.2
F	16.3	34.1
G	130.5	170.9
H	139.6	110.4

The MCF-7 or HeLa cells (2 × 10^3^ cells /well) were cultured with different ATE-Ea subfractions (6.25, 12.5, 25, 50 and 100 μg/mL) for 72 h and the IC50 was calculated from the measurement of cell proliferation using an MTT assay.

**Table 3:** The effects of AHE-Ea-K, AHE-Ea-L, ATE-Ea-E and ATE-Ea-F on the percentage of MCF-7 and Hela cells in different cell cycle phases (G0/G1, S and G2/M) ^1,2^.

	MCF-7			HeLa		
AHE-Ea-K (μg/mL)	G0/G1	S	G2/M	G0/G1	S	G2/M
0	46.9 ± 0.6 ^b^	26.8 ± 0.1 ^a^	26.4 ± 0.7 ^a^	42.9 ±1.5 ^a^	34.0 ± 2.4 ^d^	23.2 ± 10 ^a^
25	62.8 ± 0.1 ^ab^	16.8 ± 0.2 ^b^	20.4 ± 0.1 ^a^	42.7 ± 3.6 ^a^	44.3 ± 1.5 ^c^	13.0 ± 1.6 ^ab^
50	69.0 ± 1.0 ^a^	9.9 ± 0.3 ^d^	21.1 ± 1.3 ^a^	30.2 ± 1.5 ^b^	50.1 ± 3.4 ^b^	19.7 ± 4.9 ^ab^
75	72.4 ± 3.2 ^a^	13.4 ± 0.2 ^c^	14.2 ± 3.0 ^a^	36.0 ± 5.5 ^ab^	61.8 ± 6.9 ^a^	2.2 ± 1.8 ^b^
**AHE-Ea-L (μg/mL)**	G0/G1	S	G2/M	G0/G1	S	G2/M
0	47.3 ± 0.7 ^c^	32.2 ± 2.0 ^a^	20.4 ± 1.2 ^a^	56.2 ± 5.5 ^a^	24.7 ± 5.8 ^c^	19.2 ± 2.9 ^a^
25	60.0 ± 2.0 ^b^	18.8 ± 1.5 ^b^	21.2 ± 0.7 ^a^	59.7 ± 8.7 ^a^	30.4 ± 3.8 ^c^	9.9 ± 8.3 ^b^
50	63.7 ± 1.7 ^b^	14.9 ± 0.2 ^b^	21.4 ± 2.0 ^a^	38.7 ± 1.7 ^b^	41.1 ± 0.5 ^b^	20.2 ± 2.1 ^a^
75	76.1 ± 4.3 ^a^	4.0 ± 2.5 ^c^	19.9 ± 6.7 ^a^	39.4 ± 2.5 ^b^	59.3 ± 1.6 ^a^	1.3 ± 0.9 ^c^
**ATE-Ea-E (μg/mL)**	G0/G1	S	G2/M	G0/G1	S	G2/M
0	43.4 ± 2.9 ^c^	45.9 ± 1.2 ^a^	10.7 ± 1.8 ^a^	35.2 ± 6.3 ^c^	53.7 ± 8.0 ^a^	11.1 ± 3.9 ^a^
25	49.7 ± 8.6 ^b^	40.0 ± 9.3 ^b^	10.4 ± 4.7 ^a^	40.2 ± 7.1 ^b^	47.6 ± 5.6 ^ab^	12.2 ± 1.6 ^a^
50	56.1 ± 0.6 ^a^	42.8 ± 0.6 ^a^	1.1 ± 1.2 ^b^	47.7 ± 1.9 ^a^	44.6 ± 0.9 ^bc^	7.7 ± 2.8 ^a^
75	58.3 ± 1.4 ^a^	39.1 ± 0.6 ^a^	2.6 ± 0.8 ^b^	47.7 ± 1.2 ^a^	39.3 ± 0.8 ^c^	13.0 ± 2.0 ^a^
**ATE-Ea-F (μg/mL)**	G0/G1	S	G2/M	G0/G1	S	G2/M
0	51.6 ± 5.0 ^c^	36.5 ± 5.9 ^a^	11.9 ± 3.0 ^a^	42.1 ± 9.6 ^c^	42.4 ± 8.5 ^a^	15.3 ± 3.0 ^c^
25	51.4 ± 3.8 ^b^	37.1 ± 6.3 ^a^	11.5 ± 2.9 ^a^	50.5 ± 1.6 ^b^	36.2 ± 2.5 ^a^	13.3 ± 1.0 ^c^
50	65.3 ± 6.6 ^b^	24.1 ± 5.1 ^b^	10.6 ± 1.9 ^a^	54.1 ± 3.0 ^ab^	21.8 ± 3.7 ^b^	24.1 ± 6.4 ^a^
75	85.7 ± 0.6 ^a^	4.9 ± 2.1 ^c^	9.4 ± 1.5 ^a^	59.3 ± 2.7 ^a^	21.0 ± 2.1 ^b^	19.7 ± 0.6 ^b^

^1^ The MCF-7 or HeLa cells were cultured in MEM or DMEM media containing 0, 25, 50 or 75 μg/mL of AHE-Ea-K, AHE-Ea-L, ATE-Ea-E or ATE-Ea-F for 48 h. ^2^ Each data point is represented as the mean ± SD (*n* = 3). Values in the same column with different superscript letters were significantly different (*p* < 0.05) according to ANOVA and Duncan’s multiple range tests. Different letters (a, b, c, d) indicate significant differences between or among the groups (*p* < 0.05).

**Table 4:** The effects of 48 h of adlay extract subfractions (AHE-Ea-K, AHE-Ea-L, ATE-Ea-E and ATE-Ea-F) treatment on the activity of caspase-3 in MCF-7 and HeLa cells ^1,2^.

	MCF-7	HeLa
**AHE-Ea-K (μg/mL)**	Caspase-3 Positive Cells (%)	Caspase-3 Positive Cells (%)
0	0.55 ± 0.05 ^d^	2.82 ± 0.18 ^d^
25	51.57 ± 0.38 ^c^	29.37 ± 2.10 ^c^
50	53.96 ± 0.01 ^b^	33.81 ± 0.75 ^b^
75	57.83 ± 0.25 ^a^	37.94 ± 0.11 ^a^
**AHE-Ea-L (μg/mL)**	Caspase-3 Positive Cells (%)	Caspase-3 Positive Cells (%)
0	0.55 ± 0.05 ^d^	2.82 ± 0.18 ^d^
25	36.37 ± 0.18 ^c^	7.10 ± 0.01 ^c^
50	38.94 ± 0.57 ^b^	10.28 ± 0.47 ^b^
75	39.67 ± 0.32 ^a^	34.80 ± 0.86 ^a^
**AHE-Ea-E (μg/mL)**	Caspase-3 Positive Cells (%)	Caspase-3 Positive Cells (%)
0	0.56 ± 0.17 ^d^	2.82 ± 0.18 ^d^
25	17.95 ± 0.81 ^c^	29.06 ± 0.23 ^c^
50	34.04 ± 0.54 ^b^	34.75 ± 0.15 ^b^
75	37.35 ± 0.16 ^a^	38.76 ± 0.62 ^a^
**AHE-Ea-F (μg/mL)**	Caspase-3 Positive Cells (%)	Caspase-3 Positive Cells (%)
0	0.56 ± 0.17 ^d^	2.82 ± 0.18 ^d^
25	27.43 ± 0.19 ^c^	7.68 ± 0.23 ^c^
50	36.99 ± 0.13 ^b^	7.92 ± 0.15 ^b^
75	53.42 ± 0.35 ^a^	25.85 ± 0.62 ^a^

^1^ The MCF-7 or HeLa cells were cultured in MEM and DMEM media containing 0, 25, 50 and 75 μg/mL of AHE-Ea-K, AHE-Ea-L, ATE-Ea-E and ATE-Ea-F for 48 h. Then, the cells were stained using a caspase-3 kit and analyzed by flow cytometry. ^2^ Each data point represents the mean ± SD (*n* = 3). Values in the same column with different superscript letters were significantly different (*p* < 0.05) according to ANOVA and Duncan’s multiple range tests. The abcd group that does not share the same letter were significantly different from each another (*p* < 0.05).

## Data Availability

The data presented in this study are available upon request from the corresponding author.

## Supplementary Materials

The following supporting information can be downloaded at: https://www.mdpi.com/article/10.3390/molecules27133984/s1, Figure S1. LC-MS-MS analysis on combination of active subfractions from (A) AHE-EA (F to L) and (B) ATE-EA-F.; Table S1. Standards used in LC-MS-MS analysis.

## Sample Availability

Samples of the compounds are not available from the authors.
