# Supplementary material for: Adlay Seed (Coix lacryma-jobi L. var. Ma-yuen Stapf.) Ethanolic Extract Fractions and Subfractions Induce Cell Cycle Arrest and Apoptosis in Human Breast and Cervical Cancer Cell Lines
Source: Molecules. 2022 Jun 21;27(13):3984. doi: 10.3390/molecules27133984 (PMC9268182; doi:10.3390/molecules27133984)
Supplement: Supplementary file 1 [file molecules-27-03984-s001.zip › molecules-1775558-Suppelmentary.pdf]

Material and methods

Liquid Chromatography Mass Spectrometry (LC/MS) analysis

The adlay hull comprises 35.8% weight of Coix seeds, while testa occupies only 6.8%, respectively[1]; which means adlay hull is the main composition of Coix seeds waste. In order to investigate the chemical composition in active subfractions, AHE-EA-F to L (eluted with 30% to 80% EA/Hex) was combined to conduct LC-MS-MS analysis. Phenolic compounds that were reported to possess anti-tumor capacity and once isolated from Coix seeds were used for standards [2]. The above standards were obtained from Sigma, and the retention times and ionized molecular weights were listed in Table S1. LC-MS-MS analysis of active combined-subfraction in AHE-EA was carried out on a Waters (Waters Corp., Milford, MA, USA) system which equipped an Atlantis T3, DC18 and Hilic Silica Columns (2.1×150 mm column, 5μm), pumped with Finnigan Surveyor LC Pump Plus PDA Plus, detected by Finnigan Surveyor PDA Plus detector (wavelength from 200 to 600 nm) (Finnigan MAT, San Jose, CA, USA), and the flow rate was set as 0.2 mL/minute; and the parameters of electrospray ionization (ESI) mass detector were 30 arb of Sheath gas flow rate, 0 of aux and sweep gas flow rate, 5 KV of spray voltage, and 250 °C of capillary temperature. The mobile phase was solvent A [H_2O with 5% MeOH, 5% acetonitrile (ACN) and 0.1% formic acid] and solvent B (50% MeOH/ACN with 0.1% formic acid) gradients. The samples were initially eluted by 70% solvent A and 30% solvent B, and solvent B raised to 40% and 60% within 10 minutes each, increased to 75% within 7 minutes and continuously to 78% within 8 minutes, followed to 85% within another 5 minutes, and washed under 95% solvent B for 20 minutes. Finally, the mobile phase was adjusted to original 70% solvent A and 30% solvent B for maintenance. On the other side, LC-MS-MS analysis of active combined-subfraction in ATE-EA was performed using a Finnign MAT pump (P4000, Finnign) with an ultra-violet (UV) detector (UV2000, Finnign). Gradient elution was performed with 0.01% formic acid aqueous solution (v/v, pH=3.30, A) and 50% acetonitrile (ACN) in methanol (v/v, B) at a constant rate of 0.3 mL/minutes through an ODS-3 (250 × 4.6 mm, 5μm) reverse-phase column (Inertsil). Initial starting conditions were 20% B, 0-10 minutes; B increased from 20% to 40%, 10-30 minutes; B increased from 40% to 60%, 30-40 minutes; B increased from 60% to 75%, 40-50 minutes B increased from 75% to 78%, 50-60 minutes B increased from 78% to 80%, 60-70 minutes; B increased from 80% to 100%, and finally 70-80 minutes B decreased from 100% to 20% at original condition. The absorbance was set at 280 nm. This system was coupled with a Finnigan MAT LCQ ion trap mass spectrometer system (Finnigan MAT, San Jose, CA, USA) which was operated in the ESI mode. An aliquot of the bioactive fraction (10 mg/mL, 20 μL) was directly introduced into the column through the autosampler (Finnigan MAT AS3000) with nitrogen being used as the nebulizing and drying gas. The operating parameters used were as follows: a gas temperature of 250 °C, a spray needle voltage of 5 kV, a nebulizer pressure of 60 psi, and an auxiliary gas pressure of 30 psi. An ion trap containing helium damping gas was introduced following the manufacturer's protocols. The mass spectra were acquired in an m/z range of 100-1,000 with 5 microscans and a maximum ion injection time of 200 minutes. The isolated compounds were confirmed with retention time (tR) and were qualified by MS using either a negative or positive mode.

Table S1. Standards used in LC-MS-MS analysis

		AHE-EA-F to L		ATE-EA-E and F	
		molecular ion	t_R (min)	molecular ion	t_R (min)
No.	standards				
1	luteolin	$[M-H]^-$ = 285	13.92	$[M-H]^-$ = 285	12.41
2	formononetin	$[M-H]^-$ = 267	20.05	$[M-H]^-$ = 267	21.76
3	quercetin	$[M-H]^-$ = 301	14.42	$[M-H]^-$ = 301	13.56
4	homoeriodictyol	$[M-H]^-$ = 301	15.13	$[M-H]^-$ = 301	16.02
5	eriodictyol	$[M-H]^-$ = 287	12.61	$[M-H]^-$ = 287	13.11
6	apigenin	$[M-H]^-$ = 269	17.59	$[M-H]^-$ = 269	17.39
7	naringenin	$[M-H]^-$ = 271	13.52	$[M-H]^-$ = 271	15.02
8	liquiritigenin	$[M-H]^-$ = 255	11.48	$[M-H]^-$ = 255	10.22
9	isoliquiritigenin	$[M-H]^-$ = 255	20.05	$[M-H]^-$ = 255	20.34
10	chrysoeriol	$[M-H]^-$ = 269	18.41	$[M-H]^-$ = 269	17.21
11	gallic acid	$[M-H]^-$ = 169	2.71	$[M-H]^-$ = 169	2.48
12	vanillic acid	$[M+HCOOH]^-$ = 214	4.49	$[M-H]^-$ = 167	4.92
13	vanillin	$[M+HCOOH]^-$ = 198	48.97	$[M-H]^-$ = 151	54.64

(A)

(B)

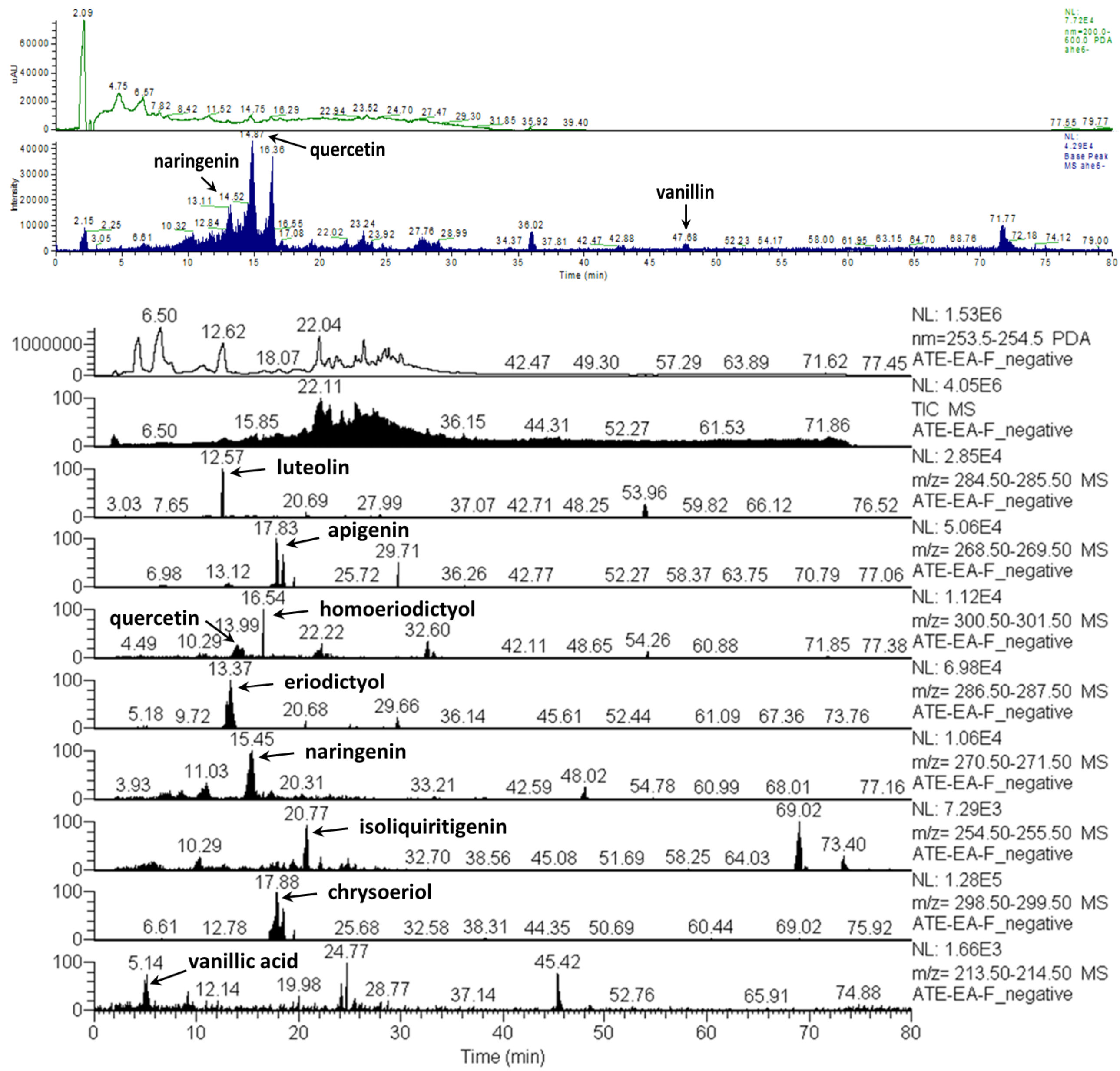

Figure S1. LC-MS-MS analysis on combination of active subfractions from (A) AHE-EA (F to L) and (B) ATE-EA-F.

1. Huang YJ, Chen YC, Chen HY, Chiang YF, Ali M, Chiang W, Chung CP, Hsia SM: **Ethanolic Extracts of Adlay Testa and Hull and Their Active Biomolecules Exert Relaxing Effect on Uterine Muscle Contraction through Blocking Extracellular Calcium Influx in Ex Vivo and In Vivo Studies.** *Biomolecules* 2021, **11**.
2. Huang YJ, Chang CC, Wang YY, Chiang WC, Shih YH, Shieh TM, Wang KL, Ali M, Hsia SM: **Adlay Testa (Coix lachryma-jobi L. var. Ma-yuen Stapf.) Ethanolic Extract and Its Active Components Exert Anti-Proliferative Effects on Endometrial Cancer Cells via Cell Cycle Arrest.** *Molecules* 2021, **26**.
